# Composition and Dynamics of H1N1 and H7N9 Influenza A Virus Quasispecies in a Co-infected Patient Analyzed by Single Molecule Sequencing Technology

**DOI:** 10.3389/fgene.2021.754445

**Published:** 2021-11-03

**Authors:** Peng Lin, Tao Jin, Xinfen Yu, Lifeng Liang, Guang Liu, Dragomirka Jovic, Zhou Sun, Zhe Yu, Jingcao Pan, Guangyi Fan

**Affiliations:** ^1^ College of Life Sciences, University of Chinese Academy of Sciences, Beijing, China; ^2^ BGI-Qingdao, BGI-Shenzhen, Qingdao, China; ^3^ BGI-Shenzhen, Shenzhen, China; ^4^ Hangzhou Center for Disease Control and Prevention, Hangzhou, China

**Keywords:** H1N1 and H7N9, quasispecies, composition and dynamics, SMRT, precision medicine

## Abstract

A human co-infected with H1N1 and H7N9 subtypes influenza A virus (IAV) causes a complex infectious disease. The identification of molecular-level variations in composition and dynamics of IAV quasispecies will help to understand the pathogenesis and provide guidance for precision medicine treatment. In this study, using single-molecule real-time sequencing (SMRT) technology, we successfully acquired full-length IAV genomic sequences and quantified their genotypes abundance in serial samples from an 81-year-old male co-infected with H1N1 and H7N9 subtypes IAV. A total of 26 high diversity nucleotide loci was detected, in which the A-G base transversion was the most abundant substitution type (67 and 64%, in H1N1 and H7N9, respectively). Seven significant amino acid variations were detected, such as NA:H275Y and HA: R222K in H1N1 as well as PB2:E627K and NA: K432E in H7N9, which are related to viral drug-resistance or mammalian adaptation. Furtherly, we retrieved 25 H1N1 and 22 H7N9 genomic segment haplotypes from the eight samples based on combining high-diversity nucleotide loci, which provided a more concise overview of viral quasispecies composition and dynamics. Our approach promotes the popularization of viral quasispecies analysis in a complex infectious disease, which will boost the understanding of viral infections, pathogenesis, evolution, and precision medicine.

## Introduction

Influenza A virus (IAV) is a contagious pathogen that constantly infects many hosts, including but not limited to humans, birds, and pigs (Medina and García-Sastre, 2011). Annual influenza virus infections have significant health and economic burdens to mankind and livestock (Gordon and Reingold, 2018). IAV is a member of the *Orthomyxoviridae* family, and its genome contains eight negative-sense single-stranded RNA segments, ranging from 850 to 2,350 bp (Pleschka, 2013; Hutchinson, 2018). IAV can be subtyped as HxNy by viral surface antigens hemagglutinin (HA) and neuraminidase (NA) proteins, which govern the viral lifecycle at cellular entry and release of virions (Dou et al., 2018). So far, eighteen different HA subtypes (H1-H18) and eleven different NA subtypes (N1-11) have been observed (Boktor and Hafner, 2019). There are two common IAV cellular receptors:α-2,3-Sialic acid (α-2,3-SA) and α-2,6-Sialic acid (α-2,6-SA) in hosts (Nelli et al., 2010; França et al., 2013; Byrd-Leotis et al., 2017; Chen et al., 2018a; Xu et al., 2019). The avian influenza viruses such H7N9 preferentially recognize α-2,3-SA receptors, while human influenza viruses such H1N1 has a priority to α-2,6-SA receptors (Xiong et al., 2013; de Graaf and Fouchier, 2014). The α-2,6-SA receptors are dominant in the upper respiratory tract (URT) of humans, while α-2,3-SA receptors are relatively more abundant than α-2,6-SA receptors found in the lower respiratory tract (LRT) of humans (Walther et al., 2013; Lakdawala et al., 2015; Long et al., 2019).

Influenza A viruses in a host exist as a population including thousands of virions containing closely related (but nonidentical) genomes, also called quasispecies (Lauring and Andino, 2010; Martínez et al., 2012; Watanabe et al., 2018; Bonomo et al., 2019; Domingo and Perales, 2019). These closely related genomes result from error-prone replication and frequent reassortment of influenza virus genomes (Steel and Lowen, 2014; Pauly et al., 2017). The complicated interactions (cooperativity or interference) among genomes and their productions collectively determine the biological or medical implications of a viral population such as fitness, virulence, pathogenesis, immune escape, or drug resistance (Vignuzzi et al., 2006; Sanz-Ramos et al., 2008; Aragri et al., 2016; Schuster, 2016; Perales, 2020). Therefore, it is primary to reveal the composition and dynamic of viral quasispecies to better understand viral infection, adaptation, and evolution at the level of population (Xue et al., 2017; Donohue et al., 2019; Jary et al., 2020).

Achieving thousands of full-length genomes in a viral population is decisive for quasispecies composition. Previous short-read massively parallel sequencing (MPS) projects have collected abundant consensus genomic sequences (CGSs) and single nucleotide variants (SNVs) to explore influenza virus quasispecies (Van den Hoecke et al., 2015; Ali et al., 2016; McGinnis et al., 2016). Nevertheless, the quasispecies composition is still unclear because of degenerated CGSs and scattered SNVs rooted in short reads from MPS (Schadt et al., 2010; Beerenwinkel et al., 2012; Chen et al., 2018b). The long-read single-molecule real-time sequencing (SMRT) provides an access to full-length influenza virus genomes even with a low frequency in a viral population (Ardui et al., 2018; Lui et al., 2019). The circular consensus sequencing (CCS) reads (average length 13.5 kb) produced by SMRT are 5–15 times as long as the genomic RNAs of influenza A virus, avoiding the fragmentation and assembly of genomes before and after sequencing (Wenger et al., 2019a; Van Poelvoorde et al., 2020).

The sample co-infected with two IAV subtypes is a very good opportunity to embody the advantage of SMRT in distinguishing different-subtype IAV genomic sequences and to quantify their abundances. The co-infection in avian hosts is common (29.59% in the live poultry market during 2016–2019 in China) (Bi et al., 2020). However, to our knowledge, there were only two human cases co-infected with two IAV subtypes reported in China since 2013. One case was a 15-year-old male co-infected with H7N9 and H3N2 in Jiangsu Province in April, 2013 and the other was a 58-year-old male with H7N9 and H1N1 in Zhejiang Province in January, 2014 (Zhu et al., 2013; Li et al., 2014). In this study, an 81-year-old male was diagnosed with H1N1 and H7N9 IAV by RT-PCR in Zhejiang Province in January 2016. Furtherly, the composition and dynamics of H1N1 and H7N9 IAV quasispecies in eight serial samples from this patient were revealed by SMRT, which provided a window to observe the viral quasispecies changes during the patient’s hospitalization treated with anti-viral drug oseltamivir.

## Materials and Methods

### Patient, Symptoms and Therapies

An 81-year-old male had a slight cough and chest distress on 1/12/2016 at his home in Xihu District, Hangzhou City, Zhejiang Province, China. On the morning of 1/15/2016, the symptoms worsened with a nasty cough, chest distress and fever. That afternoon, the man went to the local community hospital, where the temperature was 38.5 C, and then he was sent to the Hangzhou First People’s Hospital for medical treatment. The examination showed that the white blood cell count (WBC) was 13.4 × 10^9^/L, the percentage of neutrophils (N%) was 92.2%, and the C-reactive protein (CRP) was 110 mg/L. The chest radiographs showed an infection of the right lower lung. The mezlocillin sodium and sulbactam sodium were given for intravenous injection as an anti-infective therapy. The patient was sent to the respiratory department for hospital treatment and pneumonia was confirmed on 1/16/2016.

The next day, a PCR result from a throat swab was positive on influenza A virus and the patient was sent to infection ward for further treatment which included oseltamivir (75mg/bid) and meropenem drugs. On 1/19/2016, patient’s symptoms worsened further and chest radiographs confirmed that infections spread on both lungs. The patient received endotracheal intubation and then admitted to intensive care unit (ICU)The treatment was continued, the dose of oseltamivir was doubled (150mg/bid) and meropenem was changed to imipenem. On the same day, the patient’s RT-PCR test taken from the throat was positive on the M gene, H7 gene, N9 gene and H1 gene of influenza A virus. Next day, the patient was transferred to Hangzhou Xixi Hospital for treatment in isolation where the anti-viral oseltamivir (150mg/bid) continued to be given until 2/20/2016. Although, the symptomatic treatments such as diuresis, analgesia, vasodilation and nutritional support has been given in the Xixi hospital, the patient did not show signs of improvement, and passed away on 2/28/2016.

In the patient’s anamnesis it is stated that the patient went to local live poultry market and bought a live duck at a merchant’s site about a week before the first symptoms appeared on 1/12/2016. The live duck was slaughtered, depilated, and bellied by the merchant at his site. After returning home, the patient salted the duck. The patient had a history of hypertension and denied the history of diabetes, viral hepatitis, tuberculosis, and other diseases. There is no history of trauma, surgery, or blood transfusion. Denying any history of drug or food allergies.

### Samples and RT-PCR

Ten serial samples were collected from this patient from 1/19/2016 to 2/19/2016, including seven throat swabs and three sputa. The swabs and sputa were placed into 1 ml viral transport medium, transported to the laboratory within 24 h at 4°C, and then frozen at −80°C. Viral RNAs were extracted from samples using a RNeasy Mini Kit (QIAGEN, Germany). Identification of influenza A virus was achieved by RT-PCR using specific primers targeting the M, H7, N9, and H1 gene according to the protocol provided by WHO manual (Organization, W.H, 2002). This study was approved by the Institutional Review Board of BGI (NO.BGI-IRB 16008).

### Single-Molecule Real-Time Sequencing

Top eight samples were taken to perform single-molecule real-time sequencing (SMRT). The cDNAs were synthesized from viral RNAs by reverse transcription using Uni12 and Uni13 primers (Bi et al., 2016). The PCR was performed using a Phusion High-Fidelity PCR Kit (New England Biolabs) utilizing the barcoded influenza A virus general primers (Supplementary Table S1) (Mei et al., 2016). The concentration of PCR product was quantified by the Agilent Technologies 2,100 bioanalyzer. The two corresponding volumes of PCR products (containing equal mass of dsDNA) were mixed into one sample and quantified in the bioanalyzer again. About 2–3 μg mixed sample was used to SMRTbell library construction following the 2 kb template preparation protocol (Roberts et al., 2013b). The sequencing was performed on a PacBio RS II instrument (Pacific Biosciences, USA) with one SMRT Cell used for each library, using P6/C4 chemistry with a 4 h movie (Bull et al., 2016). SMRTbell adapter sequences were removed and circular consensus sequence (CCS) reads were achieved with SMRT Analysis v2.3 (Roberts et al., 2013a).

### Sequence Quality Control

The raw CCS reads were filtered by removing low quality reads (length<800bp, passes<5 or estimated accuracy<99.9%). The 800 bp length near the lower limit of influenza A virus genomic RNAs was used to exclude non-full-length genomic sequences. The other two criteria ensured reads with at least 99.9% estimated accuracy and necessary passes (Korlach, 2015). The sequencing error bases (frequency<0.3%) were additionally corrected to improve sequence reliability as follows: First, the remaining sequences after filtration were split into corresponding samples by 100% base match with barcodes. Then, the sequences of one sample were grouped by subtypes and genomic segments according to the sequence annotation result against an influenza virus genomes database downloaded from NCBI (https://ftp.ncbi.nih.gov/genomes/INFLUENZA) using BLASR (v5.1 with options: -bestn 1) (Chaisson and Tesler, 2012). All full-length genomic sequences of one group were aligned end to end using MUSCLE (v3.8.31 with default options) (Edgar, 2004). Following, the number and percentage of base A/C/T/G in the same nucleotide locus of genomic sequences were stated. Finally, the very low frequency base which percentage was less than 0.3% in the number of four type bases of the same nucleotide locus, was replaced with the dominant type of base with the largest proportion in this nucleotide locus.

### Diversity Index of Genomic Sequences

The diversity index (Shannon entropy) of one group sequences is calculated by the formula (Crooks and Brenner, 2004):

$$S=-100*\overset{n}{\underset{i=1}{\sum }}P_{i}*\log_{2}P_{i}$$

In which S is the Shannon entropy and $P_{i}$ is the ration of the number of one type of sequence to the number of total types of sequence in one group.

### Nucleotide Loci With High-Diversity Base Composition

In order to screen out nucleotide loci with high-diversity base composition, we stated the number and percentage of base A/C/T/G in one nucleotide locus and screened out the loci in which the percentages of at least two types of bases were more than 10%.

## Results

### Sequences Quality Control

**FIGURE 1:**
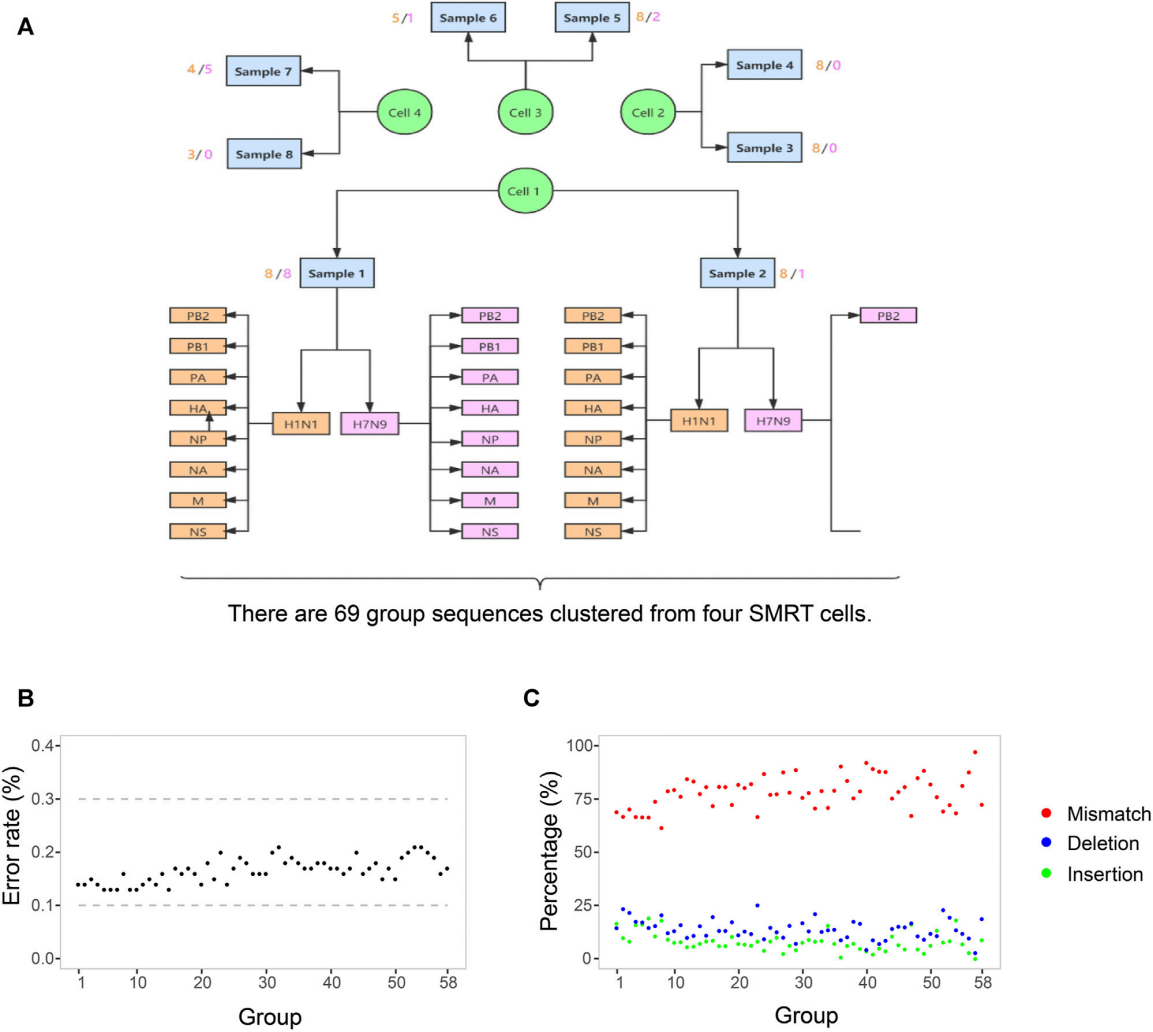
The process of sequences grouping and the SMRT sequencing error rate. **(A)** There are 69 group sequences clustered from four SMRT cells by samples, subtypes, and genomic segments. The sequences in one group indicates that they are with the same sample resource, the same Influenza subtype, and the same genomic RNA segments. The light yellow and pink figures around each sample indicate the number of groups of H1N1 and H7N9. **(B)** The base sequencing error rates of 58 group sequences. In each group, the number of sequences is more than 20. The rate is the ratio of the number of sequencing error bases to the number of total bases in one group sequences. **(C)** The three types and their percentage of sequencing error bases. red indicates mismatch, blue indicates deletion, and green indicates insertion. The percentage is the ratio of the number of one type of sequencing error bases to the number of total sequencing error bases in one group sequences. The detailed values about sequencing error bases in each group are listed in Supplementary Table S2.

The clinical symptoms and therapeutic schedule of this patient were recorded in Table 1. Ten samples were collected from this patient on different days, including seven throat swabs (S1-4, S7-10) and three sputa (S5-7). The collected date, sample types and Ct value of RT-PCR for H1N1 and H7N9 of each sample were listed in Table 2. The top eight samples (S1-8) were performed using SMRT with four SMRT cells (S9 and S10 were RT-PCR negative for influenza A virus). A total of 142,496 CCS reads (221.72 Mb) were generated from four SMRT cells, of which 82,471 high quality reads (≥99.9% estimated accuracy) were selected for further analysis according to strict filtering criteria. The IAV mutation rate was about 0.018–0.025%, which means that each replicated influenza genome (∼13 kb) contained an average of 2–3 mutations (Pauly et al., 2017). However, the estimated base sequencing error rate of high-quality CCS reads (∼0.1%) in this study, was notable higher than the normal replication mutation rate (0.018–0.025%) of IAV genomes. Therefore, it was necessary to correct sequencing error bases in the prevention of taking them for real mutations. Finally, 69 group sequences were clustered from four SMRT cells according to samples, subtypes, and genomic segments (Figure 1A). Among them, 58 group sequences were with a satisfactory abundance (the sequences number ≥20). In case that the base type (A/C/G/T) in one nucleotide locus of one group sequences was less than 0.3%, it was considered as sequencing error base. The base sequencing error rates of 58 group sequences ranged from 0.13 to 0.21%, approximate to the estimated sequencing error rate of 0.1%, which are composed of 78.37% mismatches, 13.78% deletions and 7.16% insertions (Figures 1B,C and Supplementary Table S2). Thus, we set 0.3% as the cutoff value to screen out sequencing error base types in nucleotide loci of each group sequences and correct them with dominant base types in these loci.

**TABLE 1:** The clinical symptoms and therapeutic schedule of this patient.

Date	Symptoms	Therapies
1/12/2016	Cough and chest tightness	NA
1/15/2016	Cough and chest tightness worsen, fever	The patient was sent to hospital
1/17/2016	RT-PCR positive for influenza A virus	Oseltamivir (75mg/bid) + Meropenem
1/19/2016	Lung injury was confirmed by Chest radiographs	Oseltamivir (150mg/bid) + Imipenem, Trachea cannula, ICU
1/20/2016–2/20/2016	There were no signs of improvement	Oseltamivir (150mg/bid), Symptomatic treatment (diuresis, analgesia, vasodilation, nutritional support)
2/28/2016	This patient passed away	NA

NA = Not available.

bid: Drug use frequency, twice daily.

ICU = Intensive Care Unit.

**TABLE 2:** The sampling information and RT-PCR screening of H1N1 and H7N9.

Name	Collected date	Sample type	RT-PCR screening			
			M(Ct)	H7(Ct)	N9(Ct)	H1(Ct)
S1	1/19/2016	Throat swab	+ (28)	+ (29)	+ (30)	+ (28)
S2	1/28/2016	Throat swab	+ (26)	+ (38)	+ (38)	+ (27)
S3	2/02/2016	Throat swab	+ (30)	+ (38)	+ (38)	+ (30)
S4	2/03/2016	Throat swab	+ (30)	−	−	+ (31)
S5	2/04/2016	Sputum	+ (28)	+ (36)	+ (37)	+ (29)
S6	2/05/2016	Sputum	+ (29)	−	−	+ (31)
S7	2/06/2016	Sputum	+ (28)	+ (31)	+ (31)	+ (29)
S8	2/12/2016	Throat swab	+ (31)	−	−	+ (31)
S9	2/16/2016	Throat swab	−	−	−	−
S10	2/19/2016	Throat swab	−	−	−	−

Ct: The cycle threshold value of RT-PCR.

+: Positive RT-PCR result (0 < Ct < 40).

-: Negative RT-PCR result.

### Monitoring the Composition and Dynamics of H1N1 and H7N9 Sequences

**FIGURE 2:**
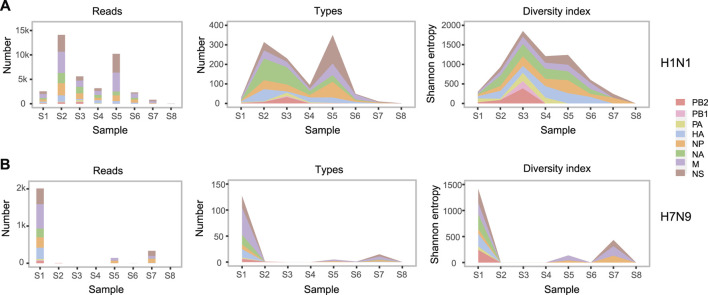
The abundance and diversity of H1N1 and H7N9 genomic sequences. In H1N1 **(A)** and H7N9 **(B)**, from left to right, three subgraphs respectively indicate the number of sequence reads, the number of sequence types and the sequence diversity index in each group. In each subgraph, eight colors represent eight genomic segments of influenza A virus. One sequence type is defined that there are one or more different bases from any other sequence. The sequence diversity index is calculated as the Shannon entropy formula described in the “Materials and Methods”. The detailed values about abundance and diversity of genomic sequences are listed in Supplementary Table S3 **.**

All the influenza virus genomic sequences produced by SMRT were clustered into 69 groups by eight samples, two subtypes, and eight genomic segments (Figure 1A). To monitor the composition and dynamic of H1N1 and H7N9 genomic sequences, we calculated the number of sequence reads, the number of sequence types, and the diversity index of sequence types in each group (Figure 2). The 16 groups (eight from H1N1 and eight from H7N9) in the first sample S1 confirmed that this patient was coinfected with H1N1 and H7N9 IAV. Interestingly, in sample S1, although the number of H1N1 and H7N9 sequence reads were almost equal, the diversity index of H7N9 sequence types was obviously higher than that of H1N1 (Figure 2). The reasons for the diversity difference of sequence types between H1N1 and H7N9 were unclear. One possible explanation was that in the upper respiratory tract (URT) environment with the dominant α-2,6-SA receptors preferentially recognized by H1N1 virus; the H7N9 virus had to generate more various genomic sequences to adapt to the relative hostile environment (Chen et al., 2013).

Furtherly, there was a sharp decrease of H7N9 viral load in the URT samples from S1 to S4. But the H7N9 viral load was still relative abundant in the subsequent LRT samples from S5 to S7 (Table 2 and Figure 2B). This might indicate that the H7N9 virus had transferred to LRT environment from URT (Gao et al., 2013). Meanwhile, there was an obvious increase of H1N1 viral load in the URT samples from S1 to S2 (Figure 2A). The reason for this increase might be due to the transfer of H7N9 from URT to LRT that contributes freeing up more cellular resources for H1N1 growth in URT environment.

### High Diversity Nucleotide Loci in H1N1 and H7N9 Genomes

Fifteen and eleven high diversity nucleotide loci were detected in H1N1 and H7N9 genomes, respectively, in which the A-G transversion was the most abundant substitution type (67% in H1N1 and 64% in H7N9) (Table 3). In H1N1, another two substitutions were C-T and A-C transversion (20 and 13% respectively). IN H7N9, the C-T, A-C and G-T took the same 0.09% proportion respectively (Table 3). In terms of amino acid, the percentage of non-synonymous mutation was greater than synonymous mutation, especially the percentage of non-synonymous mutation was up to 91% (10/11) in H7N9, comparing with the non-synonymous mutation of 66% (10/15) in H1N1(Table 3). In H1N1, all five synonymous mutations are in the internal genes of IAV, including two in NS gene (R78R and L69L), one in PB2 gene (T25T), one in NP gene (E64E) and one in M gene (R134R). In H7N9, the only one synonymous mutation was R753R in internal PB2 gene (Table 3). The H7N9 with a high frequency of non-synonymous mutation might indicate that H7N9 as avian-origin influenza virus was subjected to higher selection pressures in the human host (Wei et al., 2012; Xu et al., 2019). It is noteworthy to mention that among of these high-diversity loci, four mutations in H1N1 were involved in the evolution or viral drug-resistance, such as the R222K in the HA protein and the H275Y, A204T and K207R in NA protein (Table 3). In H7N9, three mutations were related to host adaption or viral drug-resistance, including the G685R and E627K in PB2 protein, and K432E in NA protein (Table 3).

**TABLE 3:** The high-diversity nucleotide loci in H1N1 and H7N9 genomes.

Subtype	Segment	Nucleotide loci	Base composition^a^				Amino acid^b^
			A	C	G	T	
H1N1	PB2	2045	124	0	608	0	G673D
	PB1	99	0	38	0	138	T25T
	PA	182	0	58	0	484	F53S
	HA	86	148	3,071	0	0	D18E
	HA	697	127	0	3,092	0	R222K
	NP	133	155	0	6,391	0	G30R
	NP	237	101	0	6,445	0	E64E
	NA	147	127	5,288	0	0	Q43K
	NA	630	107	0	5,308	0	A204T
	NA	640	5,280	0	135	0	K207R
	NA	843	0	4,217	0	1,198	H275Y
	M	427	663	0	11,809	0	R134R in M1
	M	835	107	0	12,365	0	W41* in M2
	NS	260	9,591	0	114	0	R78R in NS1
	NS	705	100	0	9,605	0	L69L in NEP
H7N9	PB2	1906	35	0	31	0	E627K
	PB2	2080	10	0	56	0	G685R
	PB2	2,286	52	0	14	0	R753R
	HA	722	43	0	269	0	G234D
	HA	1,163	208	0	104	0	Q381R
	NP	661	76	0	0	385	F206I
	NA	339	165	76	0	0	R107S
	NA	448	215	0	26	0	T144A
	NA	1,312	168	0	73	0	K432E
	NS	523	0	101	0	457	L166P in NEP
	NS	707	0	0	497	61	S70I in NEP

a The number of base A/C/G/T in one nucleotide locus.

b The site and types of amino acid corresponding to base composition.

Synonymous mutations.

* : Termination codon.

### The Haplotype Analysis of H1N1 and H7N9 Virus Quasispecies

**FIGURE 3:**
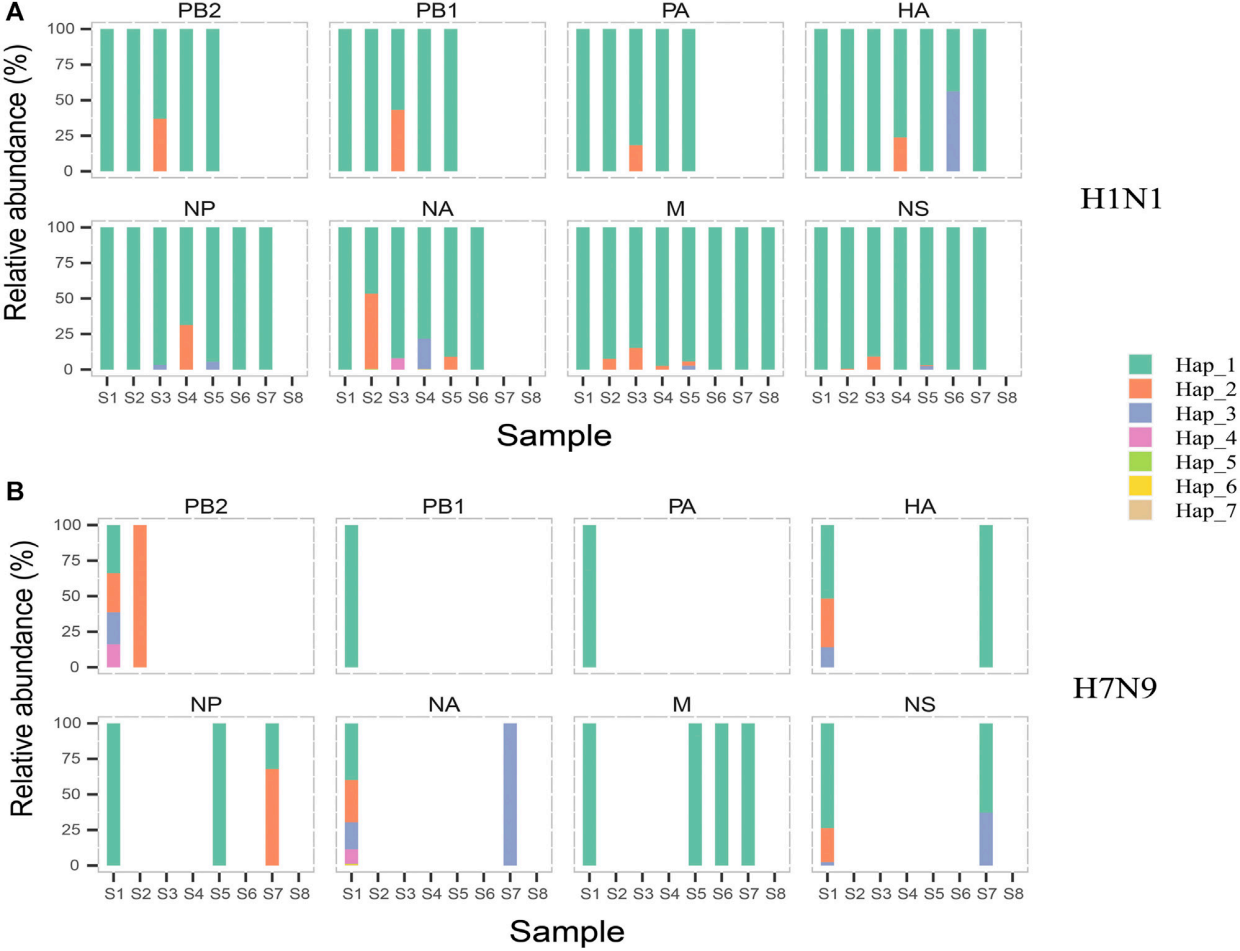
The composition and dynamics of H1N1 and H7N9 virus quasispecies. In H1N1 **(A)** or H7N9 **(B)**, eight subgraphs respectively eight genomic segments of influenza A virus. In each subgraph, different colors indicate different haplotypes. In the same subgraph, the same color represents the same haplotype, while in different subgraphs, same color is irrelevant. The forming and abundance of each haplotype are listed in Supplementary Table S4.

We achieved 25 and 22 haplotypes for H1N1 and H7N9 genomic segments in all eight samples based on combining high diversity nucleotide loci in the same genomic sequences, respectively. For instance, we obtained seven haplotypes of NA in H1N1 based on four high diversity nucleotide loci (Table 4). The bases on high diversity nucleotide loci of each haplotype and its abundance in each sample were listed in Supplementary Table S4. Then the composition and dynamics of viral quasispecies in this patient co-infected with H1N1 and H7N9 IAV were displayed along the clinical treatment process (Figure 3). In NA gene, the haplotype Hap_2 replaced Hap_1 as the dominant haplotype with more than half proportion (53.11%,1,119/2,107) in sample S2, which contain H275Y drug-resistant mutation. However, Hap_2 in NA failed to be continuously dominant and Hap_1 return the dominant haplotype in following samples (S3 to S6) (Figure 3A). Similarly, we found Hap_3 of HA with antigenic drift mutation R222K transiently become dominant in sample S6 (56.19%, 127/226) (Figure 3A). Compared with the genomic segment integrity of H1N1 viral quasispecies among almost all samples, The H7N9 quasispecies had a poor integrity except for the first sample S1 (Figure 3B). In conclusion, the haplotype provides a more concise overview of viral quasispecies composition and dynamics than whole genomic sequences (Figure 2 and Figure 3).

**TABLE 4:** The forming and abundance of NA haplotypes in H1N1.

Haplotype	Loci and bases				Abundance in each sample^a^								
	147	630	640	843	S1	S2	S3	S4	S5	S6	S7	S8	Total^b^
hap_1	C	G	A	C	282 (100%)	980 (46.51%)	1,228 (91.99%)	460 (78.23%)	710 (90.91%)	322 (100%)	0	0	3,982
hap_2	C	G	A	T	0	1,119 (53.11%)	0	0	71 (9.09%)	0	0	0	1,190
hap_3	A	G	G	C	0	0	0	126 (21.43%)	0	0	0	0	126
hap_4	C	A	A	C	0	0	107 (8.01%)	0	0	0	0	0	107
Hap_3	C	G	G	T	0	8 (0.38%)	0	0	0	0	0	0	8
hap_6	C	G	G	C	0	0	0	1 (0.17%)	0	0	0	0	1
hap_7	A	G	A	C	0	0	0	1 (0.17%)	0	0	0	0	1

a The number and percentage of haplotype in each sample.

b The number of haplotypes in all samples.

## Discussion

Four mutations in H1N1 and three in H7N9 were related to viral drug-resistance, host adaption or evolution. The H275Y in NA protein of H1N1 was the widely investigated drug-resistant mutation against oseltamivir as the commonly used first-line drug for the treatment or prophylaxis of influenza (Hurt et al., 2012; Vidaña et al., 2020). Another two mutations A204T and K207R in NA protein of H1N1 were recently reported to have effects on drug-resistance and vaccine efficacy (Nandhini and Sistla, 2020; Skowronski et al., 2020). The antigenic drift mutation R222K in the HA protein was believed to play a role in virus evolution by altering receptor binding specificity (Al Khatib et al., 2019). For H7N9, the mutation E627K on PB2 is a well-characterized host adaption mutation from the avian signature Glu (E) to the mammalian-adapted signature Lys (K), which have been associated with enhanced polymerase activity, high virus replication and pathogenicity in humans (Baccam et al., 2001). Besides, the mutation G685R on PB2 also help to promotes the mammalian adaptation of avian influenza virus (Baccam et al., 2003; Capitán et al., 2011). Interestingly, the mutation K432E on NA, alone or together with mutation H275Y on NA, had a significant impact on the binding pattern and affinity of oseltamivir for neuraminidase, rendering neuraminidase less susceptible (Aguirre and Manrubia, 2008).

The viral quasispecies as a viral population plays a very important role in the process of viral infection, adaption, and evolution through complex cooperative or competitive interactions (Domingo et al., 2012). A population of viruses can be partitioned into subpopulations by the genetic similarities (Baccam et al., 2001; Baccam et al., 2003). The spatial interactions of subpopulations have effects on host cell availability and defense responses (Aguirre and Manrubia, 2008; Capitán et al., 2011). A specific cooperative interaction is that mixed populations of D151 and G151 variants in H3N2 viruses grow better than pure populations of either variant, in which one subpopulation is good at entering new cells, while the other is better at exiting cells to spread the infection (Xue et al., 2016). In our case, the co-existent viral population of H1N1 and H7N9 might help to H7N9 subpopulation migration from URT to LRT and growth in LRT. The H1N1 subpopulation can grow in both URT and LRT of this patient, while H7N9 grow better in LRT than URT, which is related to the different distribution of α-2,3-SA and α-2,6-SA receptors in URT and LRT, as well as the preferential recognition of H1N1 and H7N9 with two receptors (de Graaf and Fouchier, 2014; Byrd-Leotis et al., 2017). The H7N9 subpopulation was easier to transfer to the LRT with the assistance of H1N1 subpopulation in the co-existence of H1N1 and H7N9 than that only in H7N9.

Besides, the competitive interactions among viral quasispecies are also reported (Andino and Domingo, 2015). An example is that in co-infected cells with wild-type polioviruses at a high multiplicity of infection (MOI) and drug-resistant virus at a much lower MOI, the yield of drug-resistant virus was significantly reduced to 3–7% of the output from a single infection due to the interference of chimeric capsid formation (Crowder and Kirkegaard, 2005). We detected the drug-resistant mutation H275Y in NA protein and antigenic drift mutation R222K in HA protein. But both failed to be continuously dominant in the subsequent viral quasispecies composition. A possible explanation is that the forming of the viral particles containing mutations were interfered by normal strains with a similar mechanism illustrated in above poliovirus.

The composition, complexity and dynamic of a viral quasispecies determined its biological or medical implications, such as host range, pathogenesis, and coping with selection pressure (Roedig et al., 2011; Gregori et al., 2016; Barbezange et al., 2018). In this work, the composition and dynamics of viral quasispecies in a patient co-infected with H1N1 and H7N9 influenza A virus were clearly revealed along his treatment process using the single-molecule real-time sequencing (SMRT). Compared with the flaws of consensus genomic sequences (CGSs) and single nucleotide variants (SNVs) by short-read massively parallel sequencing (MPS), the SMRT embody the obvious advantage in investigating the complex haplotype distribution of an IAV population, especially a population coexisting with two subtype IAV. Because of a human co-infected with two subtype IAV is very rare, the single patient in this study restricted the conclusions. Fortunately, the co-existence of two or more subtype IAV in wildfowls is not rare. These works studying the composition and dynamics of viral quasispecies in wildfowls co-infected with multi subtype IAV will be conducted in future. One scarcity of SMRT is the limit of detection (LOD) not good enough to detect the low abundant IAV sequences. For example, when the Ct values of RT-PCR for IAV in samples of this study were less than 30, SMRT was hard to generate IAV genomic sequences. This is also the reason why several types of sequences were not detected in some samples and only 69 rather than 128 groups to be conducted in the study. Besides, the expensive sequencing costs is another limitation. With the optimization and upgrade of SMRT in terms of limit of detection and sequencing cost, using SRMT to reveal the composition and dynamic of influenza A virus quasispecies will become a necessary method for studying viral biological behaviors and medical implications, which will boost the understanding of viral infections, pathogenesis, evolution, and precision medicine (Nakano et al., 2017; Wenger et al., 2019b; Beaulaurier et al., 2020).

### Accession Number

The data that support the findings of this study have been deposited into CNGB Sequence Archive (Guo et al., 2020)of CNGBdb (Chen et al., 2020)with accession number CNP0001131.

## Data Availability

Publicly available datasets were analyzed in this study. This data can be found here: https://db.cngb.org/search/project/CNP0001131/.

## References

[B1] Aguirre J. Manrubia S. C. (2008). Effects of Spatial Competition on the Diversity of a Quasispecies. Phys. Rev. Lett. 100 (3), 038106. 10.1103/PhysRevLett.100.038106 18233044

[B2] Al Khatib H. A. Al Thani A. A. Gallouzi I. Yassine H. M. (2019). Epidemiological and Genetic Characterization of pH1N1 and H3N2 Influenza Viruses Circulated in MENA Region during 2009-2017. BMC Infect. Dis. 19 (1), 314. 10.1186/s12879-019-3930-6 30971204 PMC6458790

[B3] Ali R. Blackburn R. M. Kozlakidis Z. (2016). Next-Generation Sequencing and Influenza Virus: A Short Review of the Published Implementation Attempts. HAYATI J. Biosciences 23 (4), 155–159. 10.1016/j.hjb.2016.12.007

[B4] Andino R. Domingo E. (2015). Viral Quasispecies. Virology 479-480, 46–51. 10.1016/j.virol.2015.03.022 25824477 PMC4826558

[B5] Aragri M. Alteri C. Battisti A. Di Carlo D. Minichini C. Sagnelli C. (2016). Multiple Hepatitis B Virus (HBV) Quasispecies and Immune-Escape Mutations Are Present in HBV Surface Antigen and Reverse Transcriptase of Patients with Acute Hepatitis B. J. Infect. Dis. 213 (12), 1897–1905. 10.1093/infdis/jiw049 26908731

[B6] Ardui S. Ameur A. Vermeesch J. R. Hestand M. S. (2018). Single Molecule Real-Time (SMRT) Sequencing Comes of Age: Applications and Utilities for Medical Diagnostics. Nucleic Acids Res. 46 (5), 2159–2168. 10.1093/nar/gky066 29401301 PMC5861413

[B7] Baccam P. Thompson R. J. Fedrigo O. Carpenter S. Cornette J. L. (2001). PAQ: Partition Analysis of Quasispecies. Bioinformatics 17 (1), 16–22. 10.1093/bioinformatics/17.1.16 11222259

[B8] Baccam P. Thompson R. J. Li Y. Sparks W. O. Belshan M. Dorman K. S. (2003). Subpopulations of Equine Infectious Anemia Virus Rev Coexist *In Vivo* and Differ in Phenotype. J. Virol. 77 (22), 12122–12131. 10.1128/jvi.77.22.12122-12131.2003 14581549 PMC254257

[B9] Barbezange C. Jones L. Blanc H. Isakov O. Celniker G. Enouf V. (2018). Seasonal Genetic Drift of Human Influenza A Virus Quasispecies Revealed by Deep Sequencing. Front. Microbiol. 9, 2596. 10.3389/fmicb.2018.02596 30429836 PMC6220372

[B10] Beaulaurier J. Luo E. Eppley J. M. Uyl P. D. Dai X. Burger A. (2020). Assembly-free Single-Molecule Sequencing Recovers Complete Virus Genomes from Natural Microbial Communities. Genome Res. 30 (3), 437–446. 10.1101/gr.251686.119 32075851 PMC7111524

[B11] Beerenwinkel N. Günthard H. F. Roth V. Metzner K. J. (2012). Challenges and Opportunities in Estimating Viral Genetic Diversity from Next-Generation Sequencing Data. Front. Microbio. 3, 329. 10.3389/fmicb.2012.00329 PMC3438994 22973268

[B12] Bi Y. Chen Q. Wang Q. Chen J. Jin T. Wong G. (2016). Genesis, Evolution and Prevalence of H5N6 Avian Influenza Viruses in China. Cell Host & Microbe 20 (6), 810–821. 10.1016/j.chom.2016.10.022 27916476

[B13] Bi Y. Li J. Li S. Fu G. Jin T. Zhang C. (2020). Dominant Subtype Switch in Avian Influenza Viruses during 2016-2019 in China. Nat. Commun. 11 (1), 5909. 10.1038/s41467-020-19671-3 33219213 PMC7679419

[B14] Boktor S. W. Hafner J. W. (2019). Influenza. Available from https://www.ncbi.nlm.nih.gov/books/NBK459363/ .

[B15] Bonomo M. E. Kim R. Y. Deem M. W. (2019). Modular Epitope Binding Predicts Influenza Quasispecies Dominance and Vaccine Effectiveness: Application to 2018/19 Season. Vaccine 37 (24), 3154–3158. 10.1016/j.vaccine.2019.03.068 31060950

[B16] Bull R. A. Eltahla A. A. Rodrigo C. Koekkoek S. M. Walker M. Pirozyan M. R. (2016). A Method for Near Full-Length Amplification and Sequencing for Six Hepatitis C Virus Genotypes. BMC Genomics 17, 247. 10.1186/s12864-016-2575-8 26988550 PMC4797172

[B17] Byrd-Leotis L. Cummings R. D. Steinhauer D. A. (2017). The Interplay between the Host Receptor and Influenza Virus Hemagglutinin and Neuraminidase. Int. J. Mol. Sci. 18 (7). 10.3390/ijms18071541 PMC5536029 28714909

[B18] Capitán J. A. Cuesta J. A. Manrubia S. C. Aguirre J. (2011). Severe Hindrance of Viral Infection Propagation in Spatially Extended Hosts. PLoS One 6 (8), e23358. 10.1371/journal.pone.0023358 21912595 PMC3160299

[B19] Chaisson M. J. Tesler G. (2012). Mapping Single Molecule Sequencing Reads Using Basic Local Alignment with Successive Refinement (BLASR): Application and Theory. BMC Bioinformatics 13, 238. 10.1186/1471-2105-13-238 22988817 PMC3572422

[B20] Chen F. Z. You L. J. Yang F. Wang L. N. Guo X. Q. Gao F. (2020). CNGBdb: China National GeneBank DataBase. Yi Chuan 42 (8), 799–809. 10.16288/j.yczz.20-080 32952115

[B21] Chen J. Zhao Y. Sun Y. (2018). De Novo haplotype Reconstruction in Viral Quasispecies Using Paired-End Read Guided Path Finding. Bioinformatics 34 (17), 2927–2935. 10.1093/bioinformatics/bty202 29617936

[B22] Chen X. Liu S. Goraya M. U. Maarouf M. Huang S. Chen J.-L. (2018). Host Immune Response to Influenza A Virus Infection. Front. Immunol. 9, 320. 10.3389/fimmu.2018.00320 29556226 PMC5845129

[B23] Chen Y. Liang W. Yang S. Wu N. Gao H. Sheng J. (2013). Human Infections with the Emerging Avian Influenza A H7N9 Virus from Wet Market Poultry: Clinical Analysis and Characterisation of Viral Genome. The Lancet 381 (9881), 1916–1925. 10.1016/s0140-6736(13)60903-4 PMC7134567 23623390

[B24] Crooks G. E. Brenner S. E. (2004). Protein Secondary Structure: Entropy, Correlations and Prediction. Bioinformatics 20 (10), 1603–1611. 10.1093/bioinformatics/bth132 14988117

[B25] Crowder S. Kirkegaard K. (2005). Trans-dominant Inhibition of RNA Viral Replication Can Slow Growth of Drug-Resistant Viruses. Nat. Genet. 37 (7), 701–709. 10.1038/ng1583 15965477

[B26] de Graaf M. Fouchier R. A. M. (2014). Role of Receptor Binding Specificity in Influenza A Virus Transmission and Pathogenesis. EMBO J. 33 (8), 823–841. 10.1002/embj.201387442 24668228 PMC4194109

[B27] Domingo E. Perales C. (2019). Viral Quasispecies. Plos Genet. 15 (10), e1008271. 10.1371/journal.pgen.1008271 31622336 PMC6797082

[B28] Domingo E. Sheldon J. Perales C. (2012). Viral Quasispecies Evolution. Microbiol. Mol. Biol. Rev. 76 (2), 159–216. 10.1128/mmbr.05023-11 22688811 PMC3372249

[B29] Donohue R. C. Pfaller C. K. Cattaneo R. (2019). Cyclical Adaptation of Measles Virus Quasispecies to Epithelial and Lymphocytic Cells: To V, or Not to V. Plos Pathog. 15 (2), e1007605. 10.1371/journal.ppat.1007605 30768648 PMC6395005

[B30] Dou D. Revol R. Östbye H. Wang H. Daniels R. (2018). Influenza A Virus Cell Entry, Replication, Virion Assembly and Movement. Front. Immunol. 9, 1581. 10.3389/fimmu.2018.01581 30079062 PMC6062596

[B31] Edgar R. C. (2004). MUSCLE: a Multiple Sequence Alignment Method with Reduced Time and Space Complexity. BMC Bioinformatics 5, 113. 10.1186/1471-2105-5-113 15318951 PMC517706

[B32] França M. Stallknecht D. E. Howerth E. W. (2013). Expression and Distribution of Sialic Acid Influenza Virus Receptors in Wild Birds. Avian Pathol. 42 (1), 60–71. 10.1080/03079457.2012.759176 23391183 PMC3573863

[B33] Gao R. Cao B. Hu Y. Feng Z. Wang D. Hu W. (2013). Human Infection with a Novel Avian-Origin Influenza A (H7N9) Virus. N. Engl. J. Med. 368 (20), 1888–1897. 10.1056/NEJMoa1304459 23577628

[B34] Gordon A. Reingold A. (2018). The Burden of Influenza: a Complex Problem. Curr. Epidemiol. Rep. 5 (1), 1–9. 10.1007/s40471-018-0136-1 29503792 PMC5829127

[B35] Gregori J. Perales C. Rodriguez-Frias F. Esteban J. I. Quer J. Domingo E. (2016). Viral Quasispecies Complexity Measures. Virology 493, 227–237. 10.1016/j.virol.2016.03.017 27060566

[B36] Guo X. Chen F. Gao F. Li L. Liu K. You L. (2020). CNSA: A Data Repository for Archiving Omics Data. Database (oxford). 2020, baaa055. 10.1093/database/baaa055 32705130 PMC7377928

[B37] Hurt A. C. Hardie K. Wilson N. J. Deng Y. M. Osbourn M. Leang S. K. (2012). Characteristics of a Widespread Community Cluster of H275Y Oseltamivir-Resistant A(H1N1)pdm09 Influenza in Australia. J. Infect. Dis. 206 (2), 148–157. 10.1093/infdis/jis337 22561367 PMC3379839

[B38] Hutchinson E. C. (2018). Influenza Virus. Trends Microbiol. 26 (9), 809–810. 10.1016/j.tim.2018.05.013 29909041

[B39] Jary A. Leducq V. Malet I. Marot S. Klement-Frutos E. Teyssou E. (2020). Evolution of Viral Quasispecies during SARS-CoV-2 Infection. Clin. Microbiol. Infect. 26 (11), 1560–e4. 10.1016/j.cmi.2020.07.032 PMC7378485 32717416

[B40] Korlach J. (2015). Understanding Accuracy in SMRT Sequencing. Available from http://www.pacb.com/wp-content/uploads/2015/09/Perspective_UnderstandingAccuracySMRTSequencing1.pdf .

[B41] Lakdawala S. S. Jayaraman A. Halpin R. A. Lamirande E. W. Shih A. R. Stockwell T. B. (2015). The Soft Palate Is an Important Site of Adaptation for Transmissible Influenza Viruses. Nature 526 (7571), 122–125. 10.1038/nature15379 26416728 PMC4592815

[B42] Lauring A. S. Andino R. (2010). Quasispecies Theory and the Behavior of RNA Viruses. Plos Pathog. 6 (7), e1001005. 10.1371/journal.ppat.1001005 20661479 PMC2908548

[B43] Li J. Kou Y. Yu X. Sun Y. Zhou Y. Pu X. (2014). Human Co-infection with Avian and Seasonal Influenza Viruses, China. Emerg. Infect. Dis. 20 (11), 1953–1955. 10.3201/eid2011.140897 25340661 PMC4214318

[B44] Long J. S. Mistry B. Haslam S. M. Barclay W. S. (2019). Host and Viral Determinants of Influenza A Virus Species Specificity. Nat. Rev. Microbiol. 17 (2), 67–81. 10.1038/s41579-018-0115-z 30487536

[B45] Lui W.-Y. Yuen C.-K. Li C. Wong W. M. Lui P.-Y. Lin C.-H. (2019). SMRT Sequencing Revealed the Diversity and Characteristics of Defective Interfering RNAs in Influenza A (H7N9) Virus Infection. Emerging Microbes & Infections 8 (1), 662–674. 10.1080/22221751.2019.1611346 31084471 PMC6534226

[B46] Martínez M. A. Martrus G. Capel E. Parera M. Franco S. Nevot M. (2012). Quasispecies Dynamics of RNA Viruses. Viruses: Essential Agents of Life, 21–42. 10.1007/978-94-007-4899-6_2

[B47] McGinnis J. Laplante J. Shudt M. George K. S. (2016). Next Generation Sequencing for Whole Genome Analysis and Surveillance of Influenza A Viruses. J. Clin. Virol. 79, 44–50. 10.1016/j.jcv.2016.03.005 27085509

[B48] Medina R. A. García-Sastre A. (2011). Influenza A Viruses: New Research Developments. Nat. Rev. Microbiol. 9 (8), 590–603. 10.1038/nrmicro2613 21747392 PMC10433403

[B49] Mei K. Liu G. Chen Z. Gao Z. Zhao L. Jin T. (2016). Deep Sequencing Reveals the Viral Adaptation Process of Environment-Derived H10N8 in Mice. Infect. Genet. Evol. 37, 8–13. 10.1016/j.meegid.2015.10.016 26477933

[B50] Nakano K. Shiroma A. Shimoji M. Tamotsu H. Ashimine N. Ohki S. (2017). Advantages of Genome Sequencing by Long-Read Sequencer Using SMRT Technology in Medical Area. Hum. Cel 30 (3), 149–161. 10.1007/s13577-017-0168-8 PMC5486853 28364362

[B51] Nandhini P. Sistla S. (2020). Genetic Sequencing of Influenza A (H1N1) Pdm09 Isolates from South India, Collected between 2011 and 2015 to Detect Mutations Affecting Virulence and Resistance to Oseltamivir. Indian J. Med. Microbiol. 38 (3), 324–337. 10.4103/ijmm.IJMM_20_83 33154243

[B52] Nelli R. K. Kuchipudi S. V. White G. A. Perez B. B. Dunham S. P. Chang K.-C. (2010). Comparative Distribution of Human and Avian Type Sialic Acid Influenza Receptors in the Pig. BMC Vet. Res. 6, 4. 10.1186/1746-6148-6-4 20105300 PMC2832630

[B53] Organization, W.H. (2002). WHO Manual on Animal Influenza Diagnosis and Surveillance. Geneva, Switzerland.

[B54] Pauly M. D. Procario M. C. Lauring A. S. (2017). A Novel Twelve Class Fluctuation Test Reveals Higher Than Expected Mutation Rates for Influenza A Viruses. Elife. 6, e26437. 10.7554/eLife.26437 28598328 PMC5511008

[B55] Perales C. (2020). Quasispecies Dynamics and Clinical Significance of Hepatitis C Virus (HCV) Antiviral Resistance. Int. J. Antimicrob. Agents 56 (1), 105562. 10.1016/j.ijantimicag.2018.10.005 30315919

[B56] Pleschka S. (2013). Overview of Influenza Viruses. Curr. Top. Microbiol. Immunol. 370, 1–20. 10.1007/82_2012_272 23124938

[B57] Roberts R. J., Carneiro M. O. Schatz M. C. (2013a). The Advantages of SMRT Analysis v2.3 Software Release sequencing. Genome Biol. 10.1186/gb-2013-14-7-405 PMC3953343 23822731

[B58] Roberts R. J., Carneiro M. O. Schatz M. C. (2013b). The Advantages of SMRT Sequencing. Genome Biol. 14, 405. 10.1186/gb-2013-14-6-405 23822731 PMC3953343

[B59] Roedig J. V. Rapp E. Höper D. Genzel Y. Reichl U. (2011). Impact of Host Cell Line Adaptation on Quasispecies Composition and Glycosylation of Influenza A Virus Hemagglutinin. PLoS One 6 (12), e27989. 10.1371/journal.pone.0027989 22163276 PMC3233551

[B60] Sanz-Ramos M. Díaz-San Segundo F. Escarmís C. Domingo E. Sevilla N. (2008). Hidden Virulence Determinants in a Viral Quasispecies *In Vivo* . J. Virol. 82 (21), 10465–10476. 10.1128/jvi.00825-08 18715925 PMC2573215

[B61] Schadt E. E. Turner S. Kasarskis A. (2010). A Window into Third-Generation Sequencing. Hum. Mol. Genet. 19 (R2), R227–R240. 10.1093/hmg/ddq416 20858600

[B62] Schuster P. (2016). Quasispecies on Fitness Landscapes. Curr. Top. Microbiol. Immunol. 392, 61–120. 10.1007/82_2015_469 26597856

[B63] Skowronski D. M. Zou M. Sabaiduc S. Murti M. Olsha R. Dickinson J. A. (2020). Interim Estimates of 2019/20 Vaccine Effectiveness during Early-Season Co-circulation of Influenza A and B Viruses, Canada, February 2020. Euro Surveill. 25 (7). 10.2807/1560-7917.ES.2020.25.7.2000103 PMC7043051 32098644

[B64] Steel J. Lowen A. C. (2014). Influenza A Virus Reassortment. Curr. Top. Microbiol. Immunol. 385, 377–401. 10.1007/82_2014_395 25007845

[B65] Van den Hoecke S. Verhelst J. Vuylsteke M. Saelens X. (2015). Analysis of the Genetic Diversity of Influenza A Viruses Using Next-Generation DNA Sequencing. BMC Genomics 16, 79. 10.1186/s12864-015-1284-z 25758772 PMC4342091

[B66] Van Poelvoorde L. A. E. Saelens X. Thomas I. Roosens N. H. (2020). Next-Generation Sequencing: An Eye-Opener for the Surveillance of Antiviral Resistance in Influenza. Trends Biotechnol. 38 (4), 360–367. 10.1016/j.tibtech.2019.09.009 31810633

[B67] Vidaña B. Martínez-Orellana P. Martorell J. M. Baratelli M. Martínez J. Migura-Garcia L. (2020). Differential Viral-Host Immune Interactions Associated with Oseltamivir-Resistant H275Y and Wild-type H1N1 A(pdm09) Influenza Virus Pathogenicity. Viruses 12 (8). 10.3390/v12080794 PMC7472233 32721992

[B68] Vignuzzi M. Stone J. K. Arnold J. J. Cameron C. E. Andino R. (2006). Quasispecies Diversity Determines Pathogenesis through Cooperative Interactions in a Viral Population. Nature 439 (7074), 344–348. 10.1038/nature04388 16327776 PMC1569948

[B69] Walther T. Karamanska R. Chan R. W. Y. Chan M. C. W. Jia N. Air G. (2013). Glycomic Analysis of Human Respiratory Tract Tissues and Correlation with Influenza Virus Infection. Plos Pathog. 9 (3), e1003223. 10.1371/journal.ppat.1003223 23516363 PMC3597497

[B70] Watanabe Y. Arai Y. Kawashita N. Ibrahim M. S. Elgendy E. M. Daidoji T. (2018). Characterization of H5N1 Influenza Virus Quasispecies with Adaptive Hemagglutinin Mutations from Single-Virus Infections of Human Airway Cells. J. Virol. 92 (11), e02004. 10.1128/JVI.02004-17 29563293 PMC5952156

[B71] Wei K. Chen Y. Chen J. Wu L. Xie D. (2012). Evolution and Adaptation of Hemagglutinin Gene of Human H5N1 Influenza Virus. Virus Genes 44 (3), 450–458. 10.1007/s11262-012-0717-x 22286608

[B72] Wenger A. M. Peluso P. Rowell W. J. (2019a). Highly-accurate Long-Read Sequencing Improves Variantdetection and Assembly of a Human Genome *.* Nat. Biotechnol 37, 1155. 10.1038/s41587-019-0217-9 PMC6776680 31406327

[B73] Wenger A. M. Peluso P. Rowell W. J. Chang P.-C. Hall R. J. Concepcion G. T. (2019b). Accurate Circular Consensus Long-Read Sequencing Improves Variant Detection and Assembly of a Human Genome. Nat. Biotechnol. 37 (10), 1155–1162. 10.1038/s41587-019-0217-9 31406327 PMC6776680

[B74] Xiong X. Martin S. R. Haire L. F. Wharton S. A. Daniels R. S. Bennett M. S. (2013). Receptor Binding by an H7N9 Influenza Virus from Humans. Nature 499 (7459), 496–499. 10.1038/nature12372 23787694

[B75] Xu Y. Peng R. Zhang W. Qi J. Song H. Liu S. (2019). Avian-to-Human Receptor-Binding Adaptation of Avian H7N9 Influenza Virus Hemagglutinin. Cel Rep. 29 (8), 2217–2228. 10.1016/j.celrep.2019.10.047 31747596

[B76] Xue K. S. Hooper K. A. Ollodart A. R. Dingens A. S. Bloom J. D. (2016). Cooperation between Distinct Viral Variants Promotes Growth of H3N2 Influenza in Cell Culture. Elife 5, e13974. 10.7554/eLife.13974 26978794 PMC4805539

[B77] Xue Y. Wang M. J. Yang Z. T. Yu D. M. Han Y. Huang D. (2017). Clinical Features and Viral Quasispecies Characteristics Associated with Infection by the Hepatitis B Virus G145R Immune Escape Mutant. Emerg. Microbes Infect. 6 (3), e15. 10.1038/emi.2017.2 28325923 PMC5378923

[B78] Zhu Y. Qi X. Cui L. Zhou M. Wang H. (2013). Human Co-infection with Novel Avian Influenza A H7N9 and Influenza A H3N2 Viruses in Jiangsu Province, China. Lancet 381 (9883), 2134. 10.1016/S0140-6736(13)61135-6 23769236

